# Thoracic Spinal Metastasis With Hip Flexion Failure and Psoas Muscle Atrophy Successfully Improved With Radiotherapy: A Case Report

**DOI:** 10.7759/cureus.53931

**Published:** 2024-02-09

**Authors:** Satoshi Teramura, Yojiro Ishikawa, Kengo Ito, Takayuki Yamada

**Affiliations:** 1 Division of Radiology, Tohoku Medical and Pharmaceutical University, Sendai, JPN

**Keywords:** iliopsoas syndrome, bone metastasis, irradiation, radiation oncology, radiation therapy

## Abstract

When a malignant tumor infiltrates the psoas muscle, it is termed malignant psoas syndrome (MPS). We are reporting this case because the malignancy led to atrophy of the psoas muscle, and the clinical course differed from the typical presentation of MPS. A 72-year-old Japanese female with advanced sigmoid colon cancer and multiple metastases had been undergoing systemic chemotherapy for four years. She complained of severe back pain on a numeric rating scale (NRS) of 4-5, left groin pain, and hip flexion weakness. Although she could stand up, she started experiencing difficulties while walking and became reliant on a wheelchair. At the time of referral to our department, her performance status was 2. On examination, she was capable of hip adduction and abduction, and flexion was impossible on the left side and possible on the right side. Imaging revealed metastases to the 11th and 12th thoracic vertebrae, extending to the upper portion of the first lumbar vertebra, leading to atrophy of the left psoas major muscle and impairment of hip flexion. She received palliative radiation therapy (RT) of 30 Gy in 10 fractions over a period of 2 weeks. Following RT, she had grade 1 skin inflammation but no severe complications. Two weeks after RT, her pain improved (NRS 0-1) and she regained hip flexion. When hip flexion failure occurs in patients with malignant tumors, it is important to recognize that it may be caused by a tumor located near the lower thoracic or upper lumbar spine, even if the psoas muscle itself is not directly infiltrated by the tumor.

## Introduction

Malignant psoas syndrome (MPS) is a condition in which a malignant tumor metastasizes to or invades the psoas muscle, leading to symptoms such as severe pain and painful restriction of hip joint mobility [1]. MPS syndrome is an occasionally encountered disease that has been documented in several case reports and case series reports since its initial description in 1990 [2-10]. Recently, MPS has received increased attention with the publication of review articles and retrospective studies on treatment outcomes [3,11]. Since the psoas muscle is responsible for hip flexion, the quality of life (QOL) of cancer patients in the terminal stage may significantly deteriorate due to pain resulting from tumor infiltration into the psoas muscle along with impaired gait caused by limited hip movement [3,11]. Previous reports have highlighted cases that deviate from the typical presentation, including cases in which the characteristic painful fixation of the hip joint is not observed and cases with atypical symptoms [12]. In this case report, we present a case of advanced sigmoid colon cancer in a patient who experienced wheelchair dependency due to hip pain and hip flexion failure. Despite presenting findings similar to those of MPS, there was no tumor extension into the psoas muscle. This case exhibited a clinical course distinct from that of typical MPS.

## Case presentation

**Figure 1:**
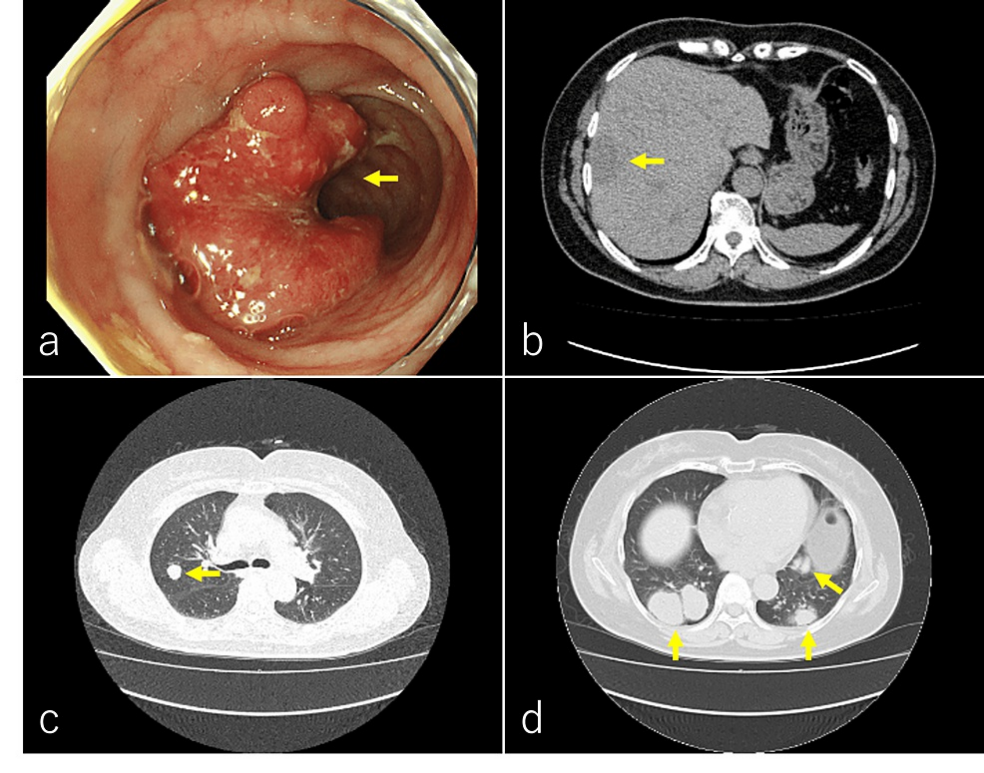
Lower endoscopy in the sigmoid colon and axial CT. Lower endoscopy revealed an elevated lesion in the sigmoid colon (a, yellow arrow). Abdominal CT showed hypodense lesions in the liver (b, yellow arrow). Lung CT showed multiple nodules in the bilateral lungs (c and d, yellow arrows).

A 72-year-old Japanese woman complained of rectal bleeding for several months. Lower gastrointestinal endoscopy revealed an elevated lesion in the sigmoid colon (Figure 1a). Pathological findings revealed adenocarcinoma. Computed tomography (CT) showed multiple metastases in the liver and bilateral lungs (Figure 1b-1d). The final diagnosis was advanced sigmoid colon cancer, cT2N0M1b, stage IVB. Her medical history included diabetes, hypertension, and dyslipidemia. The patient received systemic chemotherapy with Bev+FOLFOX (bevacizumab 5 mg/kg + oxaliplatin 85 mg/m^2^ + levofolinate 200 mg/m^2^ + fluorouracil 400 mg/m^2^) for approximately two years, which proved ineffective. This was followed by systemic chemotherapy with Bev+TAS102 (bevacizumab 5 mg/kg + trifluridine, tipiracil hydrochloride, 35 mg/m^2^) for about two years.

**Figure 2:**
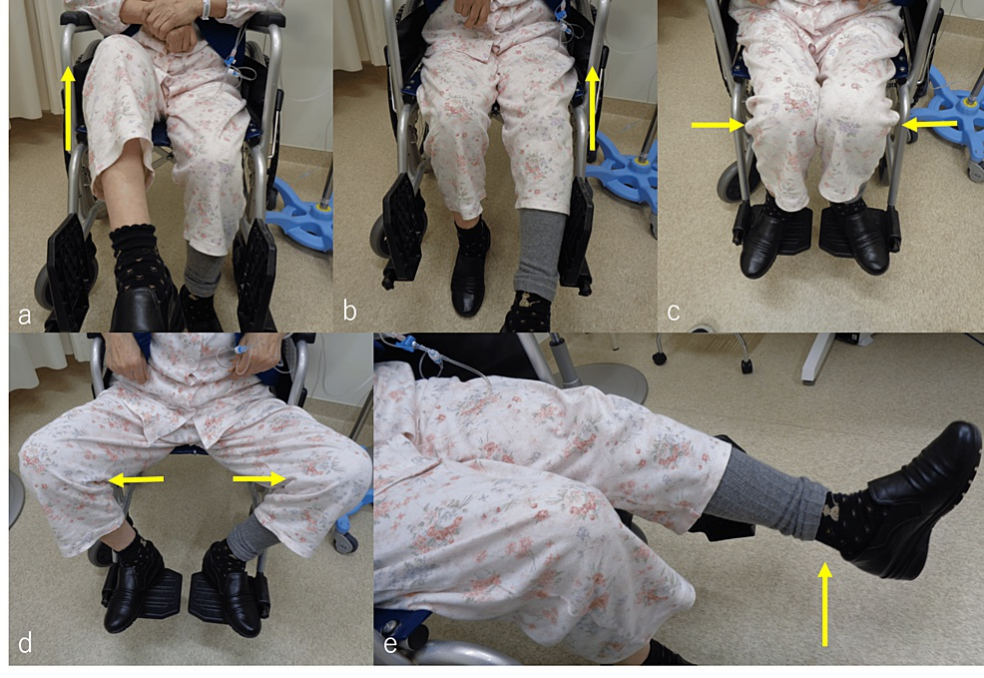
Examination findings before radiation therapy. Hip flexion was possible on the right side (a, yellow arrow) and impossible on the left side (b, yellow arrow). Hip adduction (c, yellow arrow) and abduction (d, yellow arrow) were possible on both sides. The patient was able to flex and extend her knee joint (d, yellow arrow).

Four years after the start of chemotherapy, the patient complained of severe back pain on a numeric rating scale (NRS) of 4-5, left groin pain, and hip flexion weakness for several months. Although she could stand up, she started experiencing difficulties while walking and became reliant on a wheelchair. At the time of referral to our department, her performance status was 2, as assessed by the Eastern Cooperative Oncology Group. On examination, she was capable of hip adduction and abduction, and flexion was impossible on the left side and possible on the right side. She was able to flex and extend her knee joint (Figure 2a-2e). The results of a manual muscle test (MMT) of her left hip were poor, with an MMT score of 1/5. There were no pathological reflexes or other findings indicative of spinal cord symptoms in either lower extremity. There was no painful fixation on the left hip. When transferring to a wheelchair, it was necessary for her to lift her left leg by holding it with both hands.

**Figure 3:**
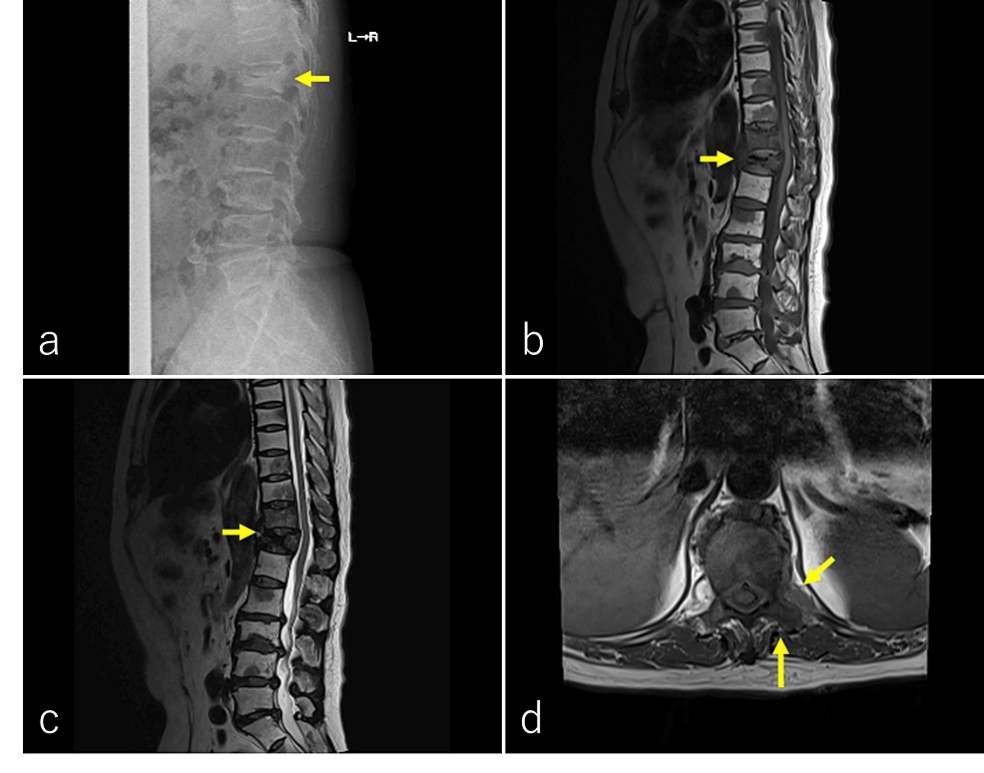
X-ray examination and vertebral magnetic resonance imaging. An X-ray examination revealed sclerotic changes and compression fracture of the 12th thoracic lumbar vertebra (a, yellow arrow). On the magnetic resonance imaging, a T1-weighted image showed low-signal findings in the 11th and 12th thoracic lumbar vertebrae and other lumbar vertebrae (b, yellow arrow). A T2-weighted image showed low-signal findings in the 11th and 12th thoracic lumbar vertebrae and other lumbar vertebrae (c, yellow arrow). A T1-weighted image showed tumor invasion on the left side from the 11th thoracic vertebra above the 1st lumbar vertebra (d, yellow arrows).

**Figure 4:**
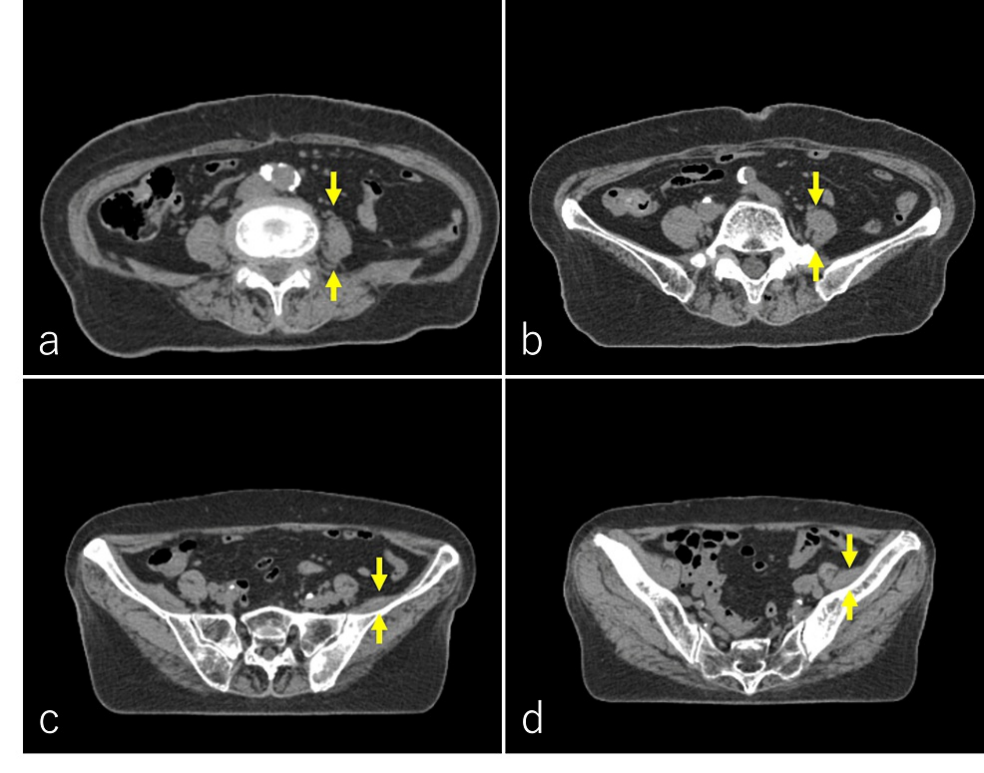
Axial pelvic CT before radiation therapy. CT images of the pelvic region revealed atrophy of the left psoas muscle compared to the right psoas muscle (a-b, yellow arrows). Some atrophy of the left iliacus muscle was also present (c-d, yellow arrows).

An X-ray examination revealed sclerotic changes and a compression fracture of the 12th thoracic lumbar vertebra (Figure 3a). T1 and T2-weighted images of magnetic resonance imaging (MRI) showed low-signal intensity in the 11th and 12th thoracic lumbar vertebras and other lumbar vertebrae. There was tumor invasion on the left side of the 11th thoracic vertebra above the 1st lumbar vertebra (Figure 3b-3d). Radiation therapy (RT) was requested for pain relief purposes. Based on examination and CT findings, the patient was diagnosed with hip flexion disorder due to atrophy of the left psoas muscle induced by the 11th and 12th thoracic lumbar vertebra and 1st lumbar vertebra metastases. In addition, her left psoas muscle was atrophied compared to that in the previous CT (Figure 4a-4d).

**Figure 5:**
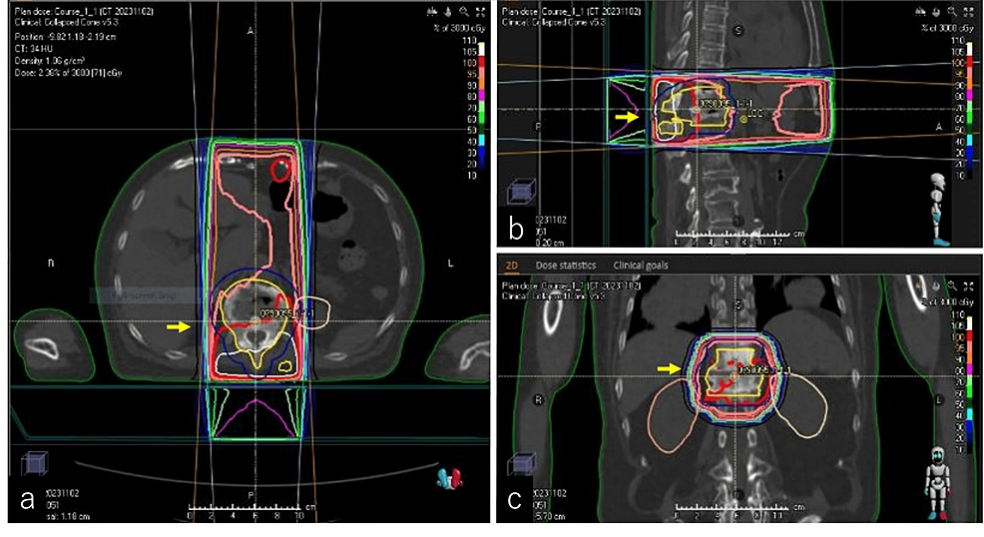
Irradiation field and dose distribution of radiation therapy. The RT plan was generated using the RayStation treatment planning system (RaySearch Laboratories AB, Stockholm, Sweden). The radiation therapy fields are displayed in axial (a, yellow arrow), sagittal (b, yellow arrow), and coronal (c, yellow arrow) images. Patient consent for treatment was obtained. We administered RT with a total dose of 30 Gy in 10 fractions over a period of two weeks. RT was delivered using 6- and 10-megavoltage equipment through a multileaf collimator by three-dimensional conformal RT from two anterior and posterior quadrants. Gross tumor volume was defined as the primary tumor in the 11th and 12th thoracic vertebrae based on the RT-planned CT. The CTV was defined as the 11th and 12th thoracic vertebrae. The planning target volume encompassed the CTV plus 1.0-cm margins, extending to include the upper part of the 1st lumbar vertebra.

We explained the risks and benefits of RT to the lumbar region, where the pain was most severe. Consent for treatment was obtained from the patient. We delivered RT of 30 Gy in 10 fractions over a period of two weeks. RT was delivered with 6- and 10-megavoltage equipment via a multileaf collimator by three-dimensional conformal RT (Figure 5a-5c). Gross tumor volume (GTV) was defined as the primary tumor in the 11th and 12th thoracic vertebrae based on the RT-planned CT. The clinical target volume (CTV) was defined as thoracic spines 11-12. The planning target volume (PTV) was CTV plus 1.0 cm margins, including above the first lumbar vertebra.

**Figure 6:**
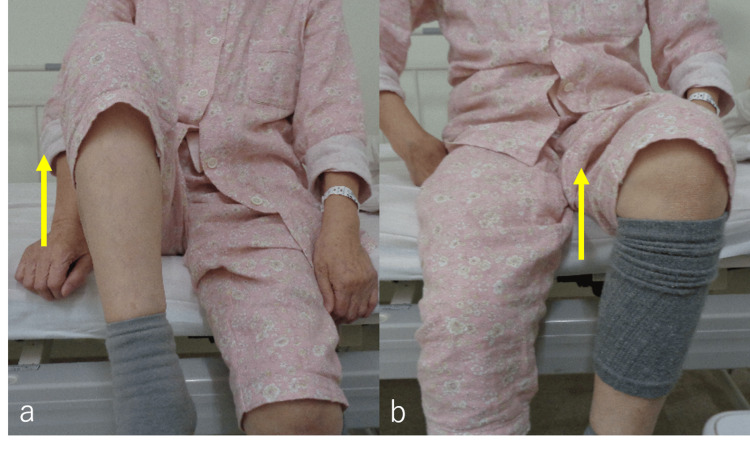
Examination findings two weeks after radiation therapy. Hip flexion was possible on the right side as before RT (a, yellow arrow). Hip flexion on the left side showed improvement compared to that before RT, and hip flexion became possible (b, yellow arrow).

An acute complication of grade 1 dermatitis (according to the National Cancer Institute Common Terminology Criteria for Adverse Events version 4.0) occurred after RT, but there was no acute complication of more than grade 2. Two weeks after the end of RT, her lower back and groin pain had improved to NRS 0-1, and she was able to flex her hip joint more than before RT (Figure 6a-6b). The results of an MMT of the left hip were fair to good, with an MMT score of 3/5 to 4/5. Because the patient died in hospice three months after RT, no follow-up imaging evaluations were conducted.

## Discussion

Malignant invasion of the psoas muscle is an occasionally encountered disease that was initially reported in 1990 [1]. MPS typically arises from distant metastasis or direct infiltration of the psoas muscle by malignancy. MPS manifests as neuropathic pain in the lumbosacral plexus region, primarily occurring during hip extension [2-11]. While MPS is commonly associated with lumbosacral plexopathy, more cases of lumbosacral plexopathy occurring in the lower lumbar spine have been reported [13]. In our patient, a tumor located between the 11th thoracic vertebra and the 1st lumbar vertebra led to dysfunction of the psoas muscle, resulting in hip flexion weakness. We have not been able to find any similar reports except for one case of lumbosacral plexopathy that appeared to be similar [13]. MPS, including lumbosacral plexopathy, leads to gait disturbances associated with hip flexion despite the absence of direct involvement of lower extremity muscles and nerves [11]. Unfortunately, despite its characteristic presentation, MPS is frequently overlooked in clinical practice, and diagnosis can be challenging due to the absence of a well-defined symptom set [3,11]. In the present case, it was difficult to link the symptom of hip flexion difficulty with the malignancy until palliative irradiation was requested, and confirmation of the diagnosis was thus delayed.

**Figure 7:**
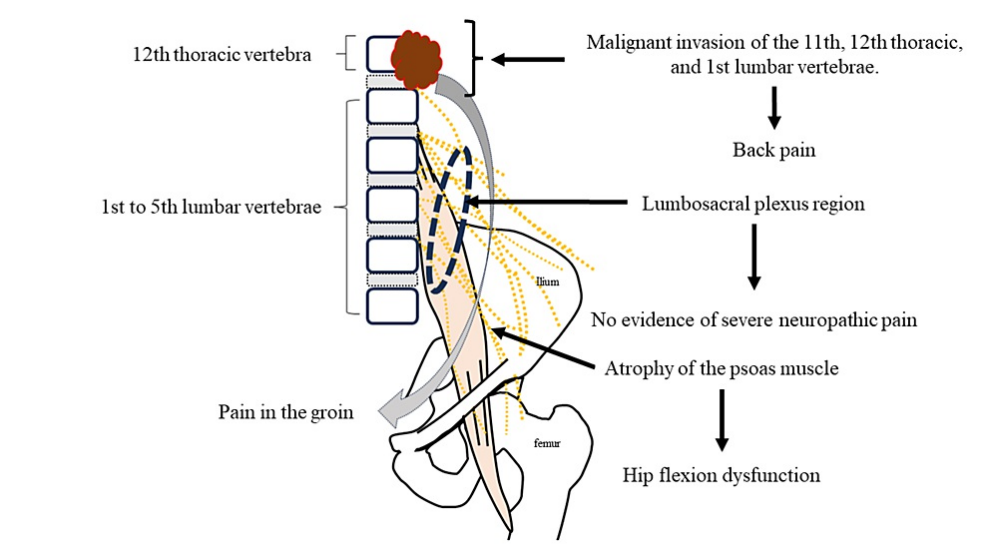
Etiological image of this Case. The metastasis of sigmoid colon cancer affected the nerve plexus at the 11th thoracic vertebra to 1st lumbar vertebra level. One distinctive feature of this case was the unilateral atrophy of the psoas muscle, which resulted in hip flexion weakness. There was no hip flexion fixation of hip pain as seen in malignant psoas syndrome because the tumor did not exert any influence on the lumbosacral plexus nerves. There was pain in the groin corresponding to the dermatome of the 11th thoracic vertebra to 1st lumbar vertebra level. Image credits: Satoshi Teramura and Yojiro Ishikawa.

The incidence of bone metastases in colorectal cancer patients has been reported to range from 1.1% to 24% [14,15]. Some reports have suggested an association between colorectal cancer and MPS [3,11,16]. Although the frequency remains uncertain, a relatively high frequency of colorectal tumors (17 of 85 cases) has also been reported in the case of lumbosacral plexopathy [13]. The pain patterns associated with MPS are generally attributed to neuropathic pain due to lumbosacral plexopathy, somatic nociceptive pain, and muscle spasms resulting from substantial psoas muscle lesions [2,3,17]. The psoas major, inguinal, and femoral inguinal nerves descend to the muscle's posterior surface behind the iliopsoas fascia [18,19]. In our patient, there was no hip pain fixation, likely due to the tumor's lack of influence on the psoas muscle and nerves (Figure 7).

One distinctive feature of this case was the unilateral psoas muscle atrophy, which led to hip flexion weakness. The course of the disease suggests that the tumor affected the nerve plexus at the T11-L1 level. Although psoas atrophy has been reported after hip joint replacement surgery [20], unilateral psoas muscle atrophy in a clinical context is exceptionally rare, and diagnosing gait disturbances caused by hip flexion weakness might be challenging. Ishikawa et al. reported exceptions in which invasion of the psoas muscle insertion site did not result in typical MPS symptoms [12]. Bone metastases near the lessor trochanter invading the psoas muscle caused hip flexion failure and difficulties in walking without painful hip flexion fixation. Our patient's symptoms in that case are similar to those in our case. As in our patient, the patient's action of elevating the hip joint by holding both legs due to voluntary hip flexion difficulty is considered a characteristic clinical manifestation of such a disease.

In our patient, palliative irradiation of bone metastases yielded pain relief and improved hip flexion. A prospective phase II study involving 60 patients with gastrointestinal cancer-related bone metastases showed pain response rates of 95-96% by radiotherapy in combination with zoledronic acid treatment [21]. Treatment options for MPS typically include analgesics and opioids, with radiation therapy being a common choice [2,3,11]. Our patient did not require high-dose, strong opioids or epidural anesthesia, and the radiotherapy was successful; the absence of intense pain of lumbosacral plexus origin, as in MPS, was characteristic of this case. Prior studies have demonstrated the efficacy of radiation therapy in managing MPS, including lumbar plexus symptoms [22-24]. Combining multiple treatment modalities, including irradiation, may be important for effective management.

## Conclusions

The symptoms in our patient improved after receiving palliative RT for unilateral psoas muscle atrophy and hip flexion failure, which were caused by bone metastatic disease affecting the 11th and 12th thoracic vertebrae resulting from sigmoid colon cancer. The extension of a malignant tumor to the psoas muscle is well recognized as MPS and is known to cause hip dysfunction, as was observed in this case. However, it is important to note that our patient did not experience the severe pain characteristic of MPS that originates from the lumbosacral plexus. When hip flexion failure occurs in patients with malignant tumors, it is important to recognize that such failure may be caused by a tumor located near the lower thoracic or upper lumbar spine, even if the psoas muscle itself is not directly infiltrated by the tumor.
